# A big fracking problem slows down a fast-swimming fish

**DOI:** 10.1093/conphys/coab004

**Published:** 2021-03-04

**Authors:** Lela S Schlenker

**Affiliations:** East Carolina University, Coastal Studies Institute, 850 NC Highway 345, Wanchese, NC 27981, USA

Mahi-mahi is one of the fastest swimming fish species in the ocean. This speedy swimming ability allows them to catch prey, avoid predators and undertake long-distance migrations in the open ocean. But, in a new study by Folkerts et al. (2020), it turns out that all of that speed is no match for the flowback wastewater that results from hydraulic fracturing, also known as ‘fracking’.

Fracking wastewater is a highly saline solution containing surfactants, heavy metals, polycyclic aromatic hydrocarbons and many other compounds and is already known to harm freshwater fishes, such as rainbow trout and zebrafish and invertebrates. While it may come as a surprise to some that not all fracking happens on land, offshore fracking is becoming increasingly common. Offshore oil and gas operations are even permitted to discharge wastewater directly into the waters of the Gulf of Mexico.

This all seems to suggest a ‘big fracking problem’ for marine fishes, such as mahi-mahi, that live in the Gulf of Mexico.

To investigate this, Folkerts and his team designed a three-part laboratory study to examine the effects of two concentrations of flowback water on mahi-mahi.

First, the researchers maintained mahi-mahi for 24 hours in aquaria containing either control seawater or flowback water. Then, they tested each fish’s maximum sustained swimming speed and found that the mahi-mahi exposed to flowback water had a top speed that was 40% slower than their control counterparts. Flowback water-exposed fish also reduced their maximum metabolic rate by 43%, which resulted in a 61% decrease in aerobic scope. Because aerobic scope represents the total amount of energy that fish have available for swimming, prey capture, predator evasion, growth and reproduction, this result indicates severe ecological impacts for flowback water-exposed mahi-mahi in the wild. Then, using a microscope, the team isolated individual heart cells from mahi-mahi and found that the flowback water-exposed cells did not contract and relax properly. Finally, the team collected tissue samples from fish from both groups and investigated the expression patterns of five cardiac genes. They found that the expression of one of the genes increased by 9-folds if the fish had been exposed to flowback water.

Folkerts’ results illustrate a cohesive story: exposure to flowback water caused cardiac abnormalities that resulted in slower-swimming mahi-mahi with less energy available for essential activities.

But, what is it about the flowback water that is so damaging? The team hypothesizes that it could be the presence of strontium and/or barium in the wastewater, as these elements could be affecting ion transport processes that are critical for the heart to function properly.

More research is needed. But in the meantime, some clear conservation implications have arisen from these results. The increase in offshore hydraulic fracturing activity and the direct release of flowback water into the marine environment stresses a critical need to document the effects of flowback water on marine life so that environmental policies can be updated to take these harmful effects into consideration before it is too late.

Section Editor: Jodie L. Rummer

Illustration by Erin Walsh; Email: ewalsh.sci@gmail.com


